# Crystallization of Electrodeposited Germanium Thin Film on Silicon (100)

**DOI:** 10.3390/ma6115047

**Published:** 2013-11-06

**Authors:** Mastura Shafinaz Zainal Abidin, Ryo Matsumura, Mohammad Anisuzzaman, Jong-Hyeok Park, Shunpei Muta, Mohamad Rusop Mahmood, Taizoh Sadoh, Abdul Manaf Hashim

**Affiliations:** 1 Faculty of Electrical Engineering, Universiti Teknologi Malaysia, Johor, Skudai 81310, Malaysia; 2 Department of Electronics, Kyushu University, 744 Motooka, Fukuoka 819-0395, Japan; E-Mails: r_matsumura@nano.ed.kyushu-u.ac.jp (R.M.); m_anisu@nano.ed.kyushu-u.ac.jp (M.A.); j_park@nano.ed.kyushu-u.ac.jp (J.-H.P.); s_muta@nano.ed.kyushu-u.ac.jp (S.M.); sadoh@ed.kyushu-u.ac.jp (T.S.); 3 Faculty of Electrical Engineering, Universiti Teknologi MARA, Selangor, Shah Alam 40450, Malaysia; E-Mail: nanouitm@gmail.com; 4 Malaysia-Japan International Institute of Technology, Universiti Teknologi Malaysia, Jalan Semarak, Kuala Lumpur 54100, Malaysia; 5 MIMOS Berhad, Technology Park Malaysia, Kuala Lumpur 57000, Malaysia

**Keywords:** germanium, silicon, electrochemical deposition, rapid melting

## Abstract

We report the crystallization of electrodeposited germanium (Ge) thin films on n-silicon (Si) (100) by rapid melting process. The electrodeposition was carried out in germanium (IV) chloride: propylene glycol (GeCl_4_:C_3_H_8_O_2_) electrolyte with constant current of 50 mA for 30 min. The measured Raman spectra and electron backscattering diffraction (EBSD) images show that the as-deposited Ge thin film was amorphous. The crystallization of deposited Ge was achieved by rapid thermal annealing (RTA) at 980 °C for 1 s. The EBSD images confirm that the orientations of the annealed Ge are similar to that of the Si substrate. The highly intense peak of Raman spectra at 300 cm^−1^ corresponding to Ge-Ge vibration mode was observed, indicating good crystal quality of Ge. An additional sub peak near to 390 cm^−1^ corresponding to the Si-Ge vibration mode was also observed, indicating the Ge-Si mixing at Ge/Si interface. Auger electron spectroscopy (AES) reveals that the intermixing depth was around 60 nm. The calculated Si fraction from Raman spectra was found to be in good agreement with the value estimated from Ge-Si equilibrium phase diagram. The proposed technique is expected to be an effective way to crystallize Ge films for various device applications as well as to create strain at the Ge-Si interface for enhancement of mobility.

## 1. Introduction

It is well known that miniaturization of transistors is very helpful in order to increase the performance of the ultra-large-scale-integrated circuits (ULSIs). Nevertheless, continuous miniaturization of transistors down to nano-scale regime tends to create several problems such as gate leakage current, short channel effect, *etc.* According to the International Technology Roadmap for Semiconductors (ITRS) 2009 edition [1], new channel materials with higher carrier mobilities than silicon (Si) are promising to enhance the switching speed of complementary metal oxide semiconductor (CMOS) transistors. Over the past decade, much attention has been paid to germanium (Ge) and III-V semiconductor channels [2,3] as the candidates to fulfill such purposes. Interestingly, these materials can not only be used to fabricate high speed conventional CMOS, but also to fabricate new transistors with different operating principles, such as tunnel field effect transistor (FET) [4], and various kinds of functional devices, such as sensors [5,6], optical devices [7], detectors [8,9,10] and solar batteries [11]. As reported by Takagi *et al.* [3], co-integration of these functional materials on Si platform seems to offer the present ULSIs with superb multi-functionalities. As a result, growth of single crystalline Ge and III-V materials on Si is becoming a key issue towards the realization of advanced heterogeneous integration on the Si platform.

In this paper, we present the crystallization of electrodeposited Ge on n-Si (100) by applying rapid melting process. In general, the deposition of polycrystalline or amorphous Ge film on Si can be performed by several techniques, such as chemical vapor deposition (CVD) and molecular beam epitaxy (MBE) system. However, these deposition techniques require ultra high vacuum environment and are expensive. On the other hand, low vacuum techniques such as sputtering and thermal or electron beam evaporation are not able to prevent the surface from being contaminated during the deposition which can affect the subsequent achievement of perfect crystallization of Ge. The electrochemical process is considered to be a promising method since it is low cost, simple and gives high deposition rate, which should be beneficial and practical.

There were a few studies that reported the electrodeposition of semiconductor thin films [12,13]. In the electrodeposition technique, electrolytes or medium solvents play an important role in determining the quality of deposited thin films. An electrodeposition of semiconductor materials has been mainly achieved in non-aqueous solvents [12] such as ionic liquids [14,15,16] and glycols [17,18]. A relatively thick Ge film can be obtained from a non-aqueous bath of a mixture of germanium (IV) chloride (GeCl_4_) or germanium (IV) iodide (GeI_4_) in ethylene glycol. However, Szekely [17] reported that electrodeposition using this ethylene glycol bath shows some difficulties in operation, *i.e.*, high corrosion rate of an anode and eventual precipitation of a white crystalline substance on the substrate. In contrast, no difficulty of operation was found by using a propylene glycol (C_3_H_8_O_2_) bath which enables a deposition of Ge thin film with better controllability of thickness [17]. The first attempt for electrodeposition of Ge from a solution of GeCl_4_ in C_3_H_8_O_2_ was done by Szekely on metal substrates, such as Ni and Cu, at several temperatures ranging from 20 to 80 °C [17,18]. Saitou *et al.* [19] deposited crystalline Ge thin films electrochemically on Cu substrates from GeCl_4_ in C_3_H_8_O_2_ electrolyte at room temperature. Instead of GeCl_4_, Endres *et al.* [14,15] reported that Ge films could also be obtained from germanium bromide (GeBr_4_) by adding suitable solvents. From these reported works, it is proven that an electrodeposition technique can be used to deposit Ge thin films on conductive substrates, including semiconductors. To our knowledge, no experimental work has yet been done by using GeCl_4_ in C_3_H_8_O_2_ electrolyte to deposit Ge films on semiconductor substrates. In this paper, we report for the first time the electrodeposition of Ge films on Si substrates by using a mixture of GeCl_4_ and C_3_H_8_O_2_.

In the Ge electrodeposition process, the important factor that has to be considered is that the deposited Ge layer should be highly pure without any excessive contamination from other elements such as oxygen. Such contamination will prevent Ge from being crystallized due to the incorporation of oxygen on the Si surface. In general, the as-deposited Ge film by any technique tends to show either amorphous or polycrystalline structure. The crystallization of amorphous Ge has been shown to be achievable by applying a so-called rapid melting process [20,21,22,23,24,25,26]. This paper presents the crystallized characteristics of electrodeposited Ge on Si by a rapid melting process.

## 2. Experimental

The electrodeposition was carried out on n-type phosphorus-doped Si (100) substrates with thickness of 355–405 μm and resistivity of 0.7–1.3 Ω cm. Prior to deposition process, the samples were cleaned by standard RCA process and buffered hydrofluoric (BHF) acid to remove native oxide layer. The electrodeposition was carried out in a simple teflon cell by using two terminal configuration where Si substrate was placed at the bottom to act as a cathode and Pt wire was used as an anode. The electrolyte is a mixture of 5% GeCl_4_ in C_3_H_8_O_2_ [19]. The process was done at room temperature with constant current of 50 mA (current density, *J* = 20 mA·cm^−2^) for 30 min in a nitrogen-filled glove box in order to minimize the effects from oxygen and moisture. After electrodeposition, the samples were immediately immersed in deionized water.

The deposited Ge films were patterned into circular islands with diameter, ϕ of 3, 5, 7, 10, 15 and 20 μm using photolithography and wet etching. A capping layer of SiO_2_/SiN*_x_* with thickness of 2 μm was deposited by magnetron sputtering. The purpose of reducing the size of Ge film and introducing capping layer is to avoid severe agglomeration of Ge during the rapid melting process [20,25]. The sample was heat treated by rapid thermal annealing (RTA) at 980 °C for 1 s to realize the crystallization of Ge. The capping layer was removed by wet etching prior to measurement. The samples were characterized using Nomarski microscopy (BX51M, Olympus Corp., Tokyo, Japan), atomic force microscopy (AFM, XE-100, Park Systems Inc., Santa Clara, CA, USA), energy dispersive X-ray spectroscopy (EDS, SU8030, Hitachi, Tokyo, Japan), Raman spectroscopy (Horiba Jobin Yvon HR640, Nd:YVO_4_ crystal, 532 nm wavelength, spot size ~1 μmϕ, Horiba, Kyoto, Japan), Auger electron spectroscopy (AES, JAMP-9500F, JEOL Ltd., Tokyo, Japan) field emission Auger microprobe, accelerating voltage: 10 kV) and electron backscattering diffraction (EBSD, EBSP JSM-5510LS scanning electron microscopy, JEOL Ltd.).

## 3. Results and Discussion

**Figure 1:**
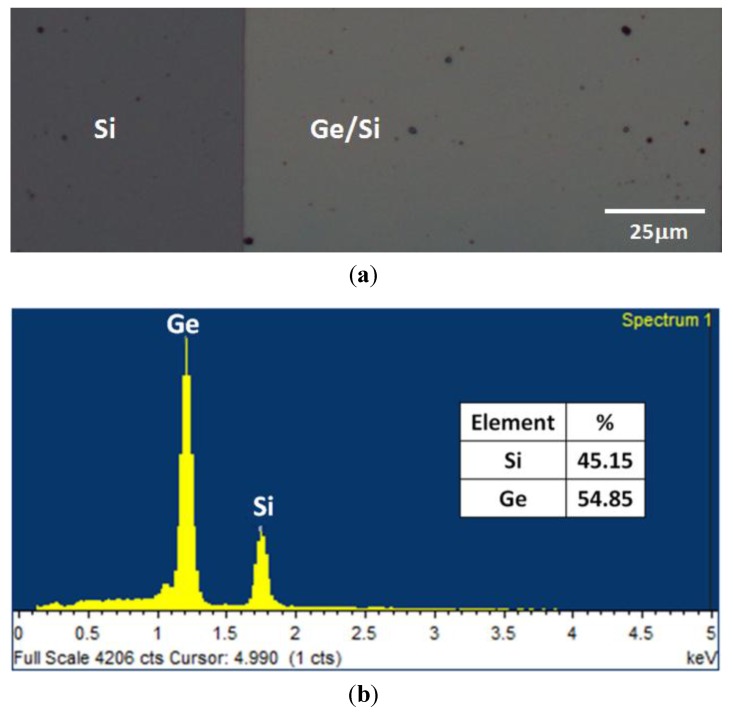

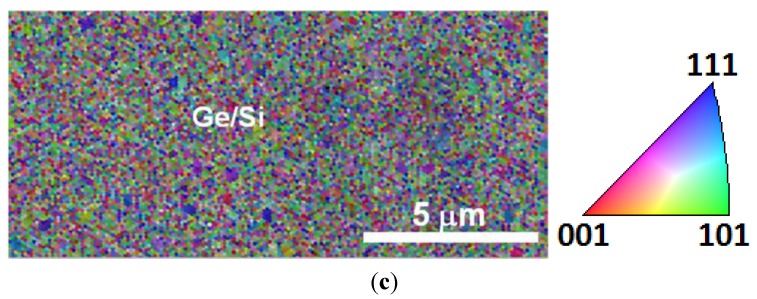
(**a**) Optical microscope images, (**b**) energy dispersive X-ray spectroscopy (EDS) and (**c**) electron backscattering diffraction (EBSD) images of as-deposited Ge on Si substrate.

Figure 1a shows the optical image of electrodeposited Ge film on n-Si substrate. The electrodeposited Ge films on n-Si at a current of 50 mA (*J* = 20 mA·cm^−2^) always show silver-like bright in color. From the optical image, no specific microstructure was observed, suggesting that the deposited film was uniform. Based on EDS spectra as shown in Figure 1b, the deposited Ge film was found to be highly pure without any excessive contaminants such as oxygen and Pt metal. For this specific sample, the thickness of Ge film measured by surface profiler was around 650 nm. The thinning process was done to reduce the thickness of electrodeposited Ge using a mixture of ammonium hydroxide, NH_4_OH: hydrogen peroxide; H_2_O_2_: deionized water, H_2_O (1:7:40). This process was applied in order to minimize the agglomeration of Ge during the rapid melting process. After the thinning process, the thickness of Ge film was around 160 nm. The EBSD analysis was carried out to examine the crystallographic orientation of the as-deposited Ge films on Si substrate. As shown in Figure 1c, random distribution of colors was obtained, indicating Ge film with amorphous structure.

**Figure 2:**
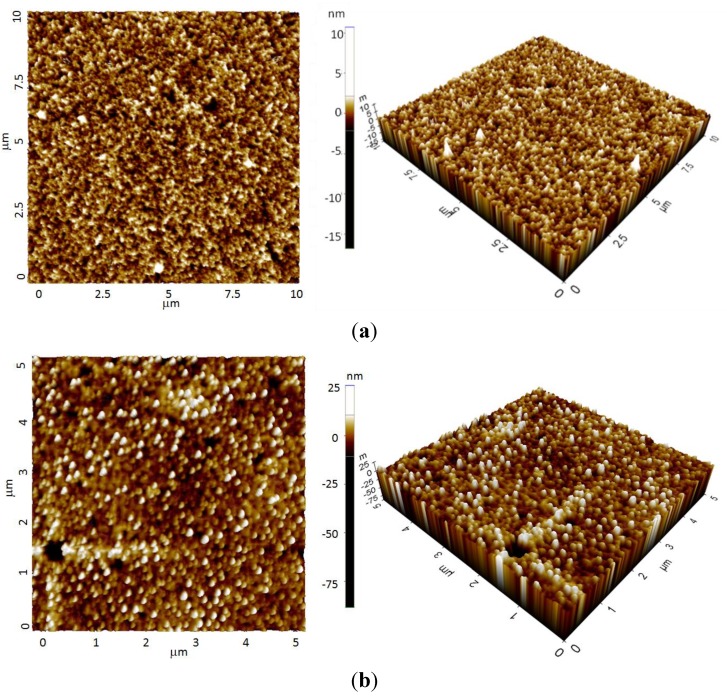
Two-dimensional (2D-) and three-dimensional (3D) atomic force microscopy (AFM) images, (**a**) as-deposited Ge/Si (Scan area = 10 μm × 10 μm, root-mean-square (RMS) = 0.828 nm) and **(b)** annealed Ge/Si (Scan area = 5 μm × 5 μm, RMS = 3.847 nm).

Further observation using AFM confirmed that uniform and relatively smooth Ge film was successfully electrodeposited on Si substrate. The two-dimensional (2D) and three-dimensional (3D) AFM images of as-deposited and annealed Ge on Si substrate were shown in Figure 2. The surface roughness in root-mean-square (RMS) was found to be 0.828 nm for as-deposited sample and slightly increased to 3.847 nm after the rapid annealing process.

**Figure 3:**
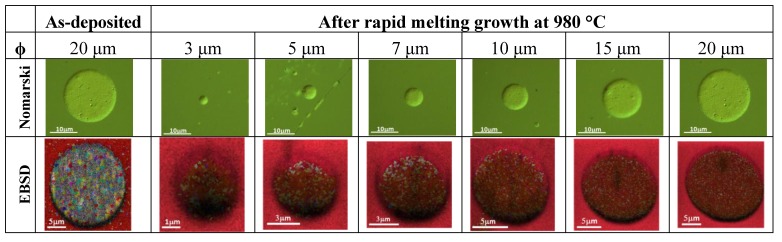
Nomarski and EBSD images of as-deposited and annealed Ge patterns.

**Figure 4:**
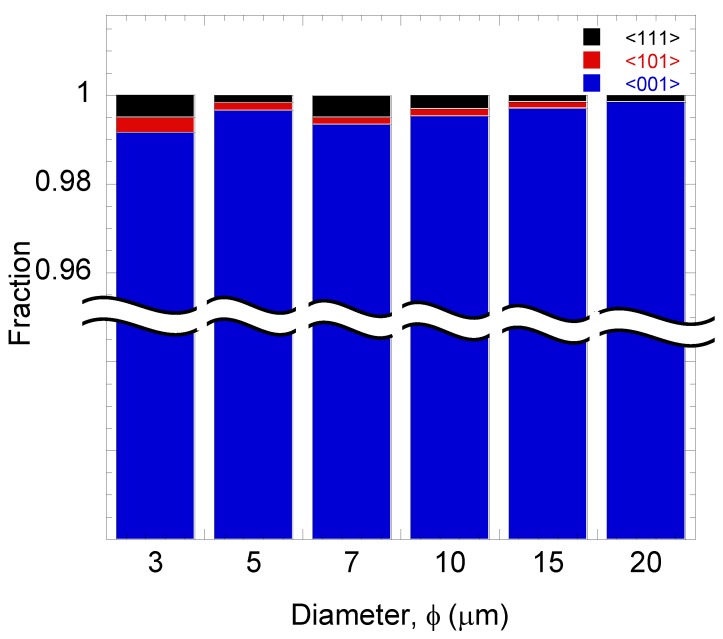
Distribution of orientation fraction of Ge patterns.

Figure 3 shows the Nomarski and EBSD images of the patterned Ge before and after the annealing process. Here, the capping layer was removed before the EBSD measurements. No agglomeration of Ge was observed, suggesting sufficient suppression by the capping layer. Based on EBSD images, the orientation of the grown Ge were confirmed to be in (100) which is similar to that of Si (100) substrate. As shown in Figure 4, the orientation mapping of the sample surface seems to be dominated by (100) with some minor orientations.

**Figure 5:**
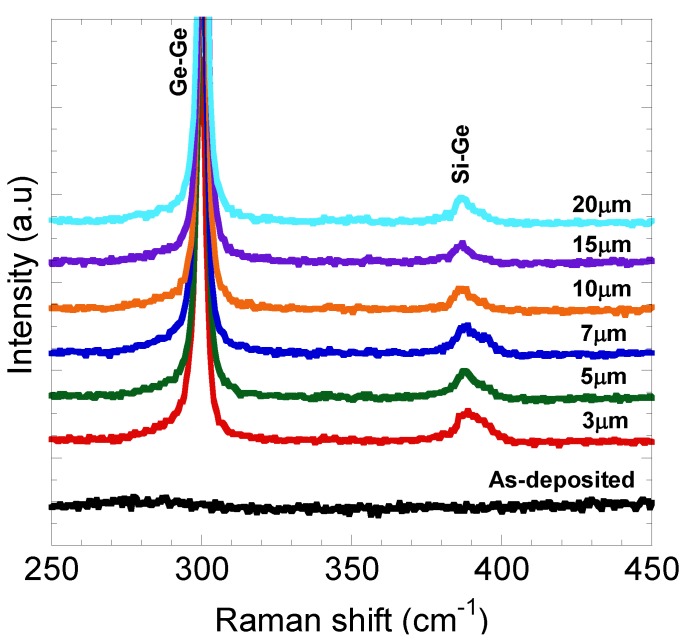
Raman spectra of as-deposited and annealed Ge patterns.

From the measured Raman spectra as shown in Figure 5, no significant Ge-Ge vibration mode peak was observed for the as-deposited Ge, thus confirming that the structure was amorphous. A similar result was also reported by Huang *et* *al*. [27] where the as-deposited Ge film deposited using 0.5 M GeCl_4_ in 1,3-propanediol electrolyte was found to be amorphous.

**Figure 6:**
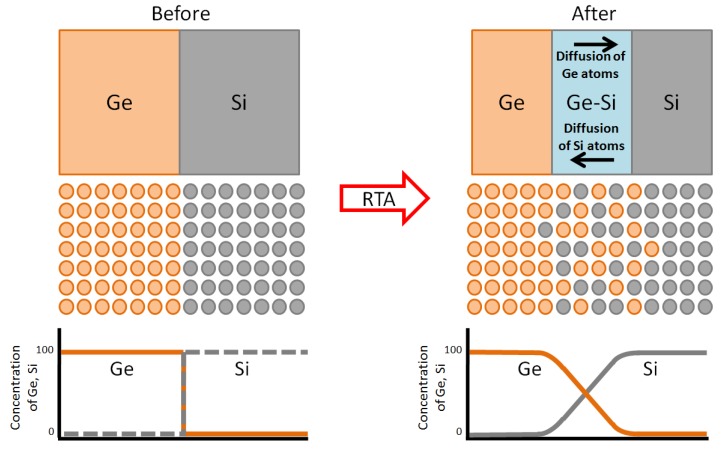
Diffusion of Si and Ge.

From Raman spectra as shown in Figure 5, the main peak at ~300 cm^−1^ which corresponds to Ge-Ge vibration mode was clearly observed after rapid annealing process, confirming the crystallization of Ge. The values of full width at half maximum (FWHM) of these peaks were estimated to be around 3.8–6.8 cm^−1^, which was slightly wider than standard single crystalline Ge wafer (3.2 cm^−1^). However, these values seem to indicate relatively good crystal quality of Ge. The sub peaks (~390 cm^−1^) due to Si-Ge vibration mode were also observed, indicating that Si-Ge mixing at Ge/Si interface occurred [25]. The diffusion of Si and Ge into each other’s regions seems to effectively take place during the melting process, as illustrated in Figure 6.

**Figure 7:**
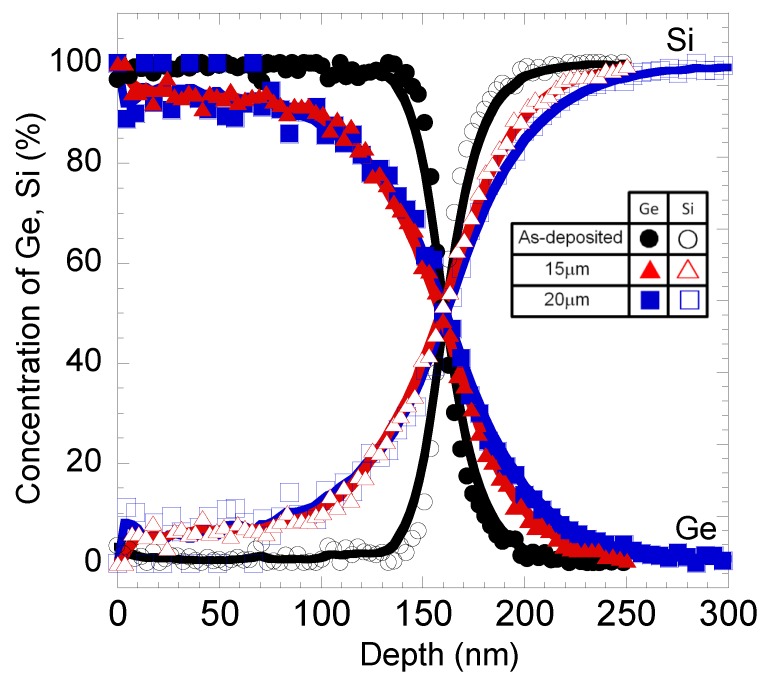
Depth profiles for sample annealed at 980 °C.

Figure 7 shows the depth profiles measured by AES for the samples (ϕ: 15 and 20 μm) before and after annealing. It is found that the intermixing layer with 60 nm thickness was formed by referring to drastic changes in the profile gradient.

The Si fraction (*x*: 0 ≤ *x* ≤ 1) in the surface regions of grown layers was calculated using Equation (1) by referring to the intensity ratio of Ge-Ge and Si-Ge vibration mode peaks obtained from Raman spectra as shown in Figure 5 [28].

(1) $$\frac{I\left(\text{Ge-Ge}\right)}{I\left(\text{Si-Ge}\right)}=\frac{k\left(1-x\right)}{2x}$$

**Figure 8:**
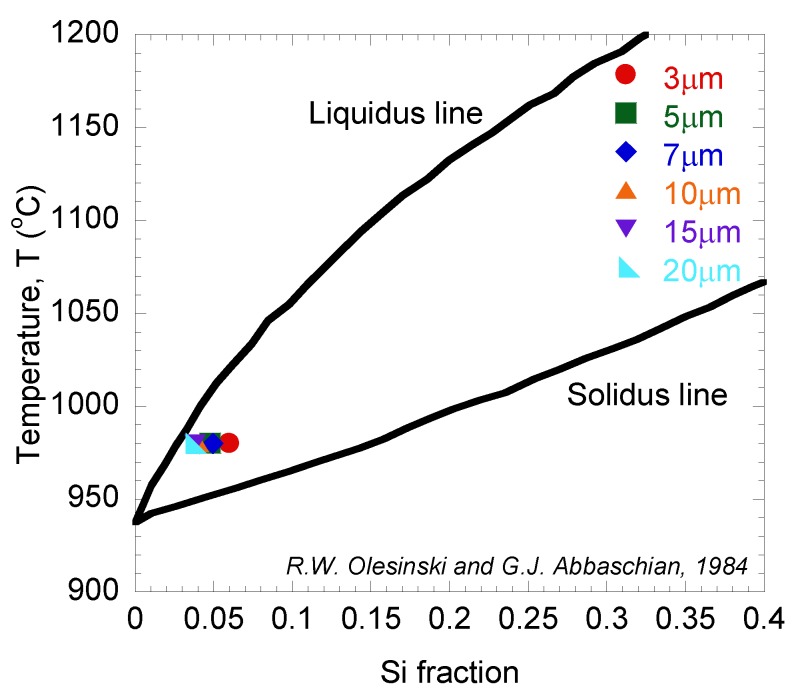
Si fraction at different ϕ, plotted in Ge-Si equilibrium phase diagram.

Here, *I*(Ge*-Ge*) and *I*(Si-Ge) are the peak intensities of Raman signals originating from Ge-Ge and Si-Ge vibration modes, respectively, and *k* is a constant. The value of *k* was determined as 1.6 which was described in [25]. As shown in Figure 8, the calculated Si fractions were plotted into the liquidus-solidus equilibrium diagram. It was found that the Si fraction was around 4% to 5% of all grown layers. These small discrepancies of Si fractions between samples with different Ge diameters could be due to the variation of latent heat distribution during the annealing process. Based on this equilibrium diagram, the points were found to be distributed near to the liquidus line [29]. The slight deviation from the liquidus line can be attributed to Si diffusion just after solidification or slight deviation in the rapid thermal annealing temperature.

For Raman spectroscopy measurement, the laser penetration depth in Ge is approximately 20 nm [30]. Therefore, these fraction values represent the amount of Si diffused into Ge layer up 20 nm depth from the surface. As shown in Figure 7, the AES depth profile of grown samples shows the clear correlation between the thickness and the amount of Si diffused into the Ge layer. It can be seen that the percentages of Si diffused into Ge layer at 20 nm depth were about 5% to 6%, for both samples with ϕ = 15 and 20 µm. These values were almost the same as the calculated values of Si fraction using Equation (1), which were found to be around 4% to 5% for the same samples.

## 4. Conclusions

In conclusion, the crystallization of electrodeposited amorphous Ge thin films on n-Si (100) by rapid melting process was successfully achieved. The intermixing region was around 60 nm from the Ge-Si interface. The proposed technique is expected to not only be an effective way to crystallize Ge films for various device applications but also to create strain at the Ge-Si interface for the enhancement of mobility.
